# Supplementary material for: Dysglycemia and the airway microbiome in cystic fibrosis
Source: PLoS One. 2025 Oct 7;20(10):e0331847. doi: 10.1371/journal.pone.0331847 (PMC12503272; doi:10.1371/journal.pone.0331847)
Supplement: S1 Table — (DOCX) [file pone.0331847.s001.docx]

**S1 Table. Differential abundance of top significant taxa comparing CFRD vs NGT**

| Microbe | Effect Estimate [95% CI] | Raw p-value | q-value | Prevalence |
| --- | --- | --- | --- | --- |
| Streptococcus.gordonii | 1.113 [0.583, 1.643] | <0.001 | 0.01 | 0.027 |
| Capnocytophaga.gingivalis | 0.518 [0.108, 0.927] | 0.018 | 0.329 | 0.022 |
| Prevotella.salivae | -0.799 [-1.440, -0.158] | 0.02 | 0.329 | 0.044 |
| Pseudomonas.aeruginosa | 2.426 [0.027, 4.824] | 0.056 | 0.449 | 0.217 |
| Streptococcus.oralis | 0.501 [-0.001, 1.004] | 0.059 | 0.449 | 0.031 |
| Haemophilus.haemolyticus | 0.479 [-0.003, 0.961] | 0.059 | 0.449 | 0.022 |
| Rothia.mucilaginosa | -1.100 [-2.258, 0.058] | 0.071 | 0.449 | 0.044 |
| Prevotella.vespertina | -0.584 [-1.224, 0.057] | 0.083 | 0.449 | 0.022 |
| Lautropia.mirabilis | -0.723 [-1.553, 0.106] | 0.097 | 0.449 | 0.027 |
| Capnocytophaga.leadbetteri | 0.318 [-0.050, 0.687] | 0.1 | 0.449 | 0.027 |
| Staphylococcus.aureus | -1.453 [-3.149, 0.244] | 0.103 | 0.449 | 0.075 |
| Streptococcus.infantis | -0.886 [-1.946, 0.174] | 0.111 | 0.449 | 0.075 |
| Prevotella.melaninogenica | -1.082 [-2.410, 0.246] | 0.12 | 0.449 | 0.119 |
| Stenotrophomonas.maltophilia | 1.254 [-0.310, 2.817] | 0.126 | 0.449 | 0.066 |
