# Supplementary material for: Dysglycemia and the airway microbiome in cystic fibrosis
Source: PLoS One. 2025 Oct 7;20(10):e0331847. doi: 10.1371/journal.pone.0331847 (PMC12503272; doi:10.1371/journal.pone.0331847)
Supplement: S2 Table — (DOCX) [file pone.0331847.s002.docx]

**S2 Table. Differential impact of CFRD on predicted functional pathways**

| KeggModules | Effect Estimate [95% CI] | q-value | Network Name | Pathway Name |
| --- | --- | --- | --- | --- |
| ko00350 | 4.541 [2.651, 6.430] | 0.002 | Metabolism | Tyr |
| ko00380 | 3.957 [2.259, 5.654] | 0.002 | Metabolism | Trp |
| ko00480 | 2.078 [0.935, 3.222] | 0.032 | Metabolism | Glutathione |
| ko01055 | -4.219 [-6.693, -1.745] | 0.036 | Metabolism | Vancomycin |
| ko02040 | 3.025 [1.250, 4.800] | 0.036 | Cellular | FlagellarAssembly |
| ko05120 | -2.895 [-4.656, -1.134] | 0.042 | Disease | HpyloriEpithelial |
| ko00660 | -4.310 [-7.055, -1.564] | 0.053 | Metabolism | C5DibasicAcid |
