# Supplementary material for: Dysglycemia and the airway microbiome in cystic fibrosis
Source: PLoS One. 2025 Oct 7;20(10):e0331847. doi: 10.1371/journal.pone.0331847 (PMC12503272; doi:10.1371/journal.pone.0331847)
Supplement: S3 Table — (DOCX) [file pone.0331847.s003.docx]

**S3 Table. Antimicrobial resistance genes abundance comparing CFRD vs NGT**

| ARO | Effect Estimate  [95% CI] | p-value | q-value | mechanism |
| --- | --- | --- | --- | --- |
| ARO 3002679 | 2.032 [0.904, 3.161] | 0.001 | 0.114 | Chloramphenicol acetyltransferases |
| ARO 3000026 | -2.382 [-3.853, -0.910] | 0.003 | 0.156 | Drug and biocide MATE efflux pumps |
| ARO 3000190 | -1.598 [-2.810, -0.386] | 0.014 | 0.193 | Tetracycline resistance ribosomal protection proteins |
| ARO 3000498 | -2.663 [-4.711, -0.614] | 0.015 | 0.193 | 23S rRNA methyltransferases |
| ARO 3000802 | 1.549 [0.338, 2.760] | 0.017 | 0.193 | Multi-biocide RND efflux pump |
| ARO 3003681 | 1.442 [0.209, 2.675] | 0.028 | 0.193 | Phenolic RND efflux pump |
| ARO 3000377 | 1.582 [0.300, 2.864] | 0.021 | 0.193 | Drug and biocide RND efflux pumps |
| ARO 3000149 | 1.495 [0.328, 2.662] | 0.017 | 0.193 | Fosfomycin thiol transferases |
| ARO 3000803 | 1.275 [0.172, 2.377] | 0.030 | 0.193 | Drug and biocide RND efflux pumps |
| ARO 3005064 | 1.739 [0.272, 3.206] | 0.026 | 0.193 | Colistin phosphoethanolamine transferase |
| ARO 3002985 | 1.227 [0.167, 2.286] | 0.029 | 0.193 | Polymyxin B resistance regulator |
