# Supplementary material for: Dysglycemia and the airway microbiome in cystic fibrosis
Source: PLoS One. 2025 Oct 7;20(10):e0331847. doi: 10.1371/journal.pone.0331847 (PMC12503272; doi:10.1371/journal.pone.0331847)
Supplement: S1 File — (DOCX) [file pone.0331847.s004.docx]

**Supplementary methods**

**Nucleic acid extraction**

Briefly, 500 µL of each sputum sample and 500 µL of cetyl trimethyl ammonium bromide (CTAB) is added to individual Lysing Matrix E tubes (MP Biomedicals), incubated at 95°C for 5 minutes followed by beadbeating for three 30 second cycles at 7.0 m/s, incubated with proteinase K at 70°C for 10 minutes, 300 sample µL lysate collected, additional beadbeating for three 30 second cycles at 7.0 m/s with each cycle, and additional 300 sample µL lysate collected. Sample lysates are transferred to 96 well plates for binding, washing, and elution steps on the Kingfisher Flex sample purification system. Extracted nucleic acids were quantified using the PicoGreen assay kit (Invitrogen), library preparation was performed using the Nextera XT DNA library preparation Kit (Illumina), and the library was sequenced on the NovaSeq 6000 platform to generate 2 × 150 base pair reads.
