# Supplementary material for: Dysglycemia and the airway microbiome in cystic fibrosis
Source: PLoS One. 2025 Oct 7;20(10):e0331847. doi: 10.1371/journal.pone.0331847 (PMC12503272; doi:10.1371/journal.pone.0331847)
Supplement: Attachment — Submitted filename: PLOS_Response_to_Review_20250625.docx [file pone.0331847.s006.docx]

**We greatly appreciate the thoughtful review of our manuscript. We have made numerous revisions in response to these comments, which we believe have improved the quality of the manuscript. We describe these in detail below in bold.**

**Reviewer #1:** The authors attempt to associate cystic fibrosis related diabetes condition to changes in the airway microbiome using metagenomics which as the authors have evidenced in this study and analysis, an important factor in understanding this lung disease. The study design is sound with recruitment, sample and clinical data collection, group stratification based on glucose tolerance, etc. The statistical analysis performed is also very appropriate with the metrics used widely in ecology studies (diversity indices, species abundance, species richness, etc.). The preliminary conclusions seem to be supported by the data and overall this paper seems complete with a note in the discussion on future directions with more rigorous strategies on glycemic characterization, longitudinal follow-up, etc.

**Response: Thank you for your generous comments and suggestions.**

Only a few minor comments follow:
1. I understand the logic behind use of inverse Simpson index to assess species diversity and Bray-Curtis distance for dissimilarity in species composition was very intuitive. It would be great if the data were also tested on another diversity index since in my experience the sensitivity of true diversity to rare vs abundant species varies in different indices. Although not crucial, but this would be good to verify the conclusions of the study.

**Response:** **We appreciate this thoughtful suggestion regarding the use of additional diversity indices. We have done so and the results remain consistent. We revised the results as below:**

In our linear model assessing the relationship between glycemic status and alpha diversity, CFRD status was associated with lower alpha diversity, as reflected by a decrease in the Inverse Simpson index (-1.98 [-3.80, -0.16], p = 0.033) and a nonsignificant reduction in species richness (-3.13 [-7.61, 1.34], p = 0.17). In the multivariate model, the effect size of the association between CFRD status and the Inverse Simpson index remained similar, with a beta estimate of -2.00 [-4.02, 0.02] (p = 0.052). IGT status was not significantly associated with alpha diversity. To test the hypothesis that glycemia affected overall microbial community structure, we assessed differences in beta diversity. CFRD status was associated with microbial community structure as measured by the Bray Curtis (PERMANOVA *p*=0.011, R^2^= 17%) (**Figure 1**) and Horn-Morisita (PERMANOVA *p=* 0.0067, R^2^ = 19%) dissimilarity in the multivariate model.

2. The value of inverse Simpson indicating a lower alpha diversity associated with CFRD status in results section (-2.30) is different than the one given in the abstract (-1.98) although confidence intervals and p values agree.

**Response: We apologize for the inconsistency. The results section reported an incorrect effect size and was updated to the correct one.**

3. In Figure 4 showing Antibiotic resistance gene abundance differences, the labels overlap a lot so hard to read. Also the figure uses BH corrected q values on y axis but the figure description mentions using raw p values instead. Also a small justification of why figure 2 used raw p values whereas in 3 and 4, BH q values were used.

**Response: We have updated figure 4 to have less overlap and the updated the legend to use q-values. We have updated all the figures to use q values for consistency.**

Figure 2. Differentially abundant species in people with CFRD vs NGT . The x-axis represents the effect size (regression coefficient) for CFRD status, while the y-axis shows the negative log10-transformed q-values. Each point represents a microbial taxon, colored by phylum

4. In Figure 5a and 5b, is there a specific reason that the bars are different colors? They don't match other figures which have their own legends which makes complete sense if colors are different, but the lack of a legend in 5a and 5b suggests that the bars really don't need to be colored.

**Response: We appreciate this helpful suggestion. We agree that the color variation in Figures 5a and 5b was unnecessary and potentially confusing. We have now updated both figures to use a single, consistent color for all bars. Additionally, we have included updated loading plots that incorporate covariates for clarity and completeness. These changes have improved the consistency and interpretability of the figure.**

5A	5B

5. Table 2 which mentions association between microbiome and virome CFRD signatures and percent predicted FEV1 seems like the main table supporting the conclusion of the paper that virome and not microbiome being associated to lung function. This table was hard for me to really understand what the different regression models were in terms of which combinations of confounders were tested so a brief explanation on how models 1 through 5 are different would be nice.

**Response: Thank you for point this out. We have updated the table legend to better describe each models** **as below.**

| **Table 2. Association between microbiome and virome CFRD signatures and % predicted FEV1.** | | | | | |
| --- | --- | --- | --- | --- | --- |
|  | **Model 1** | **Model 2** | **Model 3** | **Model 4** | **Model 5** |
| Virome^a^ | -6.1 (-9.2, -3.0)*** | - | -5.6 (-8.8, -2.4)** | - | -3.8 (-7.3, -0.3)* |
| Microbiome^a^ | - | -2.9 (-6.3, 0.5) | - | -3.2 (-6.9, 0.5) | 0.6 (-2.9, 4.0) |
| Age, years | - | - | -0.2 (-0.7, 0.3) | -0.3 (-0.8, 0.4) | -0.2 (-0.6, 0.2) |
| Female sex | - | - | 3.9 (-9.7, 17.6) | 10.6 (-5.2, 26.5) | 13.2 (2.0, 24.5)* |
| CFTR Modulator | - | - | -1.0 (-16.3, 14.2) | 4.1 (-13.2, 21.4) | -2.6 (-15.7, 10.5) |
| CFRD status | - | - | -7.8 (-22.4, 6.9) | -7.1 (-24.7, 10.5) | 2.1 (-12.5, 16.6) |
| Antibiotics | - | - | -16.6 (-38.1, 4.8) | -25.3 (-52.9, 2.2) | -17.4(-33.6, -1.3)* |
| Univariate and multivariate linear regression models evaluating the association between CFRD-related microbiome and virome signatures and percent predicted FEV1. Model 1 includes only the virome score, while Model 2 includes only the microbiome score. Models 3 and 4 assess the virome and microbiome scores, respectively, with adjustment for age, sex, modulator use, CFRD status, and antibiotic use. Model 5 includes both the virome and microbiome scores in a single model, also adjusting for the same covariates. * *p* < 0.05; ** *p* < 0.01; *** *p*<0.001. ^a^ Virome and microbiome signature scores range from 0 – 100% with higher indicating more similar to CFRD and lower indicating more similar to NGT. Effect estimates provided for a 10% increase in score. | | | | | |

6. Apart from these, minor typos in the discussion sections and where Trimmomatic is cited in the methods section. Overall, a fun and exciting paper to review.

**Response: Thank you! We have fixed the typos.**
